# Pd-Catalyzed microwave-assisted synthesis of phosphonated 13α-estrones as potential OATP2B1, 17β-HSD1 and/or STS inhibitors

**DOI:** 10.3762/bjoc.14.262

**Published:** 2018-11-14

**Authors:** Rebeka Jójárt, Szabolcs Pécsy, György Keglevich, Mihály Szécsi, Réka Rigó, Csilla Özvegy-Laczka, Gábor Kecskeméti, Erzsébet Mernyák

**Affiliations:** 1 Department of Organic Chemistry, University of Szeged, Dóm tér 8, H-6720 Szeged, Hungary; 2 Department of Organic Chemistry and Technology, Budapest University of Technology and Economics, H-1521 Budapest, Hungary; 3 1st Department of Medicine, University of Szeged, Korányi fasor 8–10, H-6720 Szeged, Hungary; 4 Membrane protein research group, Institute of Enzymology, Research Centre for Natural Sciences, Hungarian Academy of Sciences, Magyar tudósok körútja 2, H-1117 Budapest, Hungary; 5 Department of Medicinal Chemistry, University of Szeged, Dóm tér 8, H-6720 Szeged, Hungary

**Keywords:** catalysis, enzyme, 13α-estrone, Hirao reaction, 17β-HSD1 inhibition, OATP2B1, STS

## Abstract

Novel 2- or 4-phosphonated 13α-estrone derivatives were synthesized via the Hirao reaction. Bromo regioisomers (2- or 4-) of 13α-estrone and its 3-benzyl or 3-methyl ether were reacted with diethyl phosphite or diphenylphosphine oxide using Pd(PPh_3_)_4_ as catalyst under microwave irradiation. The influence of the new compounds on the transport function of the organic anion transporting polypeptide OATP2B1 was investigated by measuring Cascade Blue uptake. Derivatives bearing a 3-benzyl ether function displayed substantial submicromolar OATP2B1 inhibitory activity. The inhibitory effects of the compounds on human placental steroid sulfatase (STS) and 17β-hydroxysteroid dehydrogenase type 1 isozyme (17β-HSD1) were investigated by in vitro radiosubstrate incubation methods. None of the test compounds inhibited the STS markedly. The structure–activity relationship evaluation revealed that 2-substituted 3-hydroxy derivatives are able to inhibit the 17β-HSD1 enzyme with submicromolar IC_50_ values. Dual OATP2B1 and 17β-HSD1 inhibitors have been identified.

## Introduction

The biosynthesis of estrogens occurs via various enzymatic routes. Cytochrome P450 aromatase catalyzes the conversion of nonaromatic steroids to estrogens [1]. Moreover, hydrolysis of estrone 3-sulfate, existing as a large circulatory reservoir catalyzed by steroid sulfatase (STS), is one of the major alternative sources of prehormone estrone for the local supply of estrogens [2]. The availability of free estrone on the cellular level depends on the expression of STS and/or SULT, which catalyze the sulfation of the phenolic hydroxy function. On the other hand, estrone sulfate is not able to cross the cell membrane passively; therefore, a carrier is needed to mediate its transport across the lipid bilayer. These carriers are certain representatives of the solute carrier superfamily (SLC), including members of the organic anion transporting polypeptides (OATP) protein family [3–4]. Human OATP2B1 is one of the OATPs transporting estrone 3-sulfate, expressed in the intestine, blood–brain barrier, liver and placenta [5–9]. Moreover, OATP2B1 is overexpressed in certain malignancies, including breast cancer [10]. OATP2B1 transports its substrates, including estrone-sulfate in a sodium- and ATP-independent manner [11–12]. After entering the cells, estrone-3-sulfate is subjected to enzymatic conversions involving different cytosolic enzymes. The desulfation catalyzed by STS is followed by stereospecific reduction of the 17-oxo function leading to 17β-estradiol. This last step of the estrogen biosynthesis is catalyzed by the 17β-hydroxy steroid dehydrogenase type 1 isoenzyme (17β-HSD1) [13]. Thus STS and 17β-HSD1 became important drug targets in estrogen-dependent diseases [1,14]. Their inhibition could be a powerful strategy for the suppression of local estrogen production. Additionally, the entry of estrone-sulfate into the cells by inhibition of OATPs can be a good alternative [15–16]. Prior suppression of cytosolic enzymes involved in the estradiol biosynthesis, the transport of conjugated estrone derivatives, including estrone-3-sulfate could be blocked. Inhibitors based on the estrane core could have multiple inhibitory properties concerning the two enzymatic steps of the sulfatase pathway and OATP2B1-mediated membrane transport of estrone-sulfate.

The inhibitor design is usually based on the substrate of the target protein. The literature reveals diverse synthetic estrone derivatives as STS or 17β-HSD1 inhibitors [1,14], but to the best of our knowledge, there are no reports on estrone-based OATP2B1 inhibitors. Note that estrone itself has been shown to slightly inhibit estrone-3-sulfate uptake by OATP2B1 [6]. None of the steroidal STS or 17β-HSD1 inhibitors reached the clinical trial, which is mainly due to their retained estrogenic activity. This side-effect could be eliminated by the inhibitor design based on the 13-epimer of natural estrone (13α-estrone, 13αE1OH) [17–18]. Poirier et al. proved that 13α-derivatives of 3,17-estradiols have reduced binding affinity for estrogen receptor alpha and display no uterotropic activity [19]. The conformational change resulting from the inversion at C-13 leads to an epimer unable to bind to its nuclear receptors. We have recently established that 13α-estrone, irrespective of the substantial structural changes, displays an affinity to 17β-HSD1 similar to that of the natural substrate estrone [20]. This finding led us to design novel 17β-HSD1 inhibitors based on the hormonally inactive 13α-estrane core. Ring A of 13α-estrone was transformed in order to get 2-substituted derivatives [21–23]. On the basis of the crystal structure of 17β-HSD1 in its complex with 17β-estradiol, Möller et al. described, that there is an unoccupied lipophilic tunnel located near the C-2 atom of the steroid [24]. Their strategy for improving the inhibitory activity of natural estrone derivatives was the introduction of large halogens and phenethynyl or phenethyl functions onto C-2 of estrone. Their most potent compounds displayed nanomolar inhibitory activities. Our related previous work included the synthesis and in vitro 17β-HSD1 determination of similar derivatives of the hormonally inactive 13α-estrane core [21–23]. We found that 2- and/or 4-substitution with large halogens (Br or I) lead to submicromolar inhibitors irrespective of the position and number of the introduced halogens. In the case of phenethynyl- or phenethyl-13α-estrones, only 2-regioisomers displayed substantial inhibitory action, which did not depend on the hybrid state of carbon attached to C-2 [22]. Phan et al. described that halogenation of ring A of estrone is a powerful strategy in the synthesis of effective STS inhibitors [25]. Certain 4-halogenated estrone derivatives displayed higher affinity to the enzyme than that of their parent compound estrone. Based on these findings, we tested our halogenated 13α-estrone derivatives against STS, too [23]. Both the nature and the position of the introduced halogen influenced the STS inhibitory potential markedly. The presence of large substituents in 4-iodo and 2,4-diiodo compounds proved to be advantageous, since these compounds inhibited the enzyme with IC_50_ values in the low micromolar range. It can be stated that introduction of large lipophilic groups onto C-2 or C-4 of 13α-estrone might improve their affinity to 17β-HSD1 and STS, respectively. In order to get more structure–activity data concerning the effect of the nature and the position of the introduced functional group onto inhibitory activity, the synthesis and testing of novel ring A substituted 13α-estrone derivatives would be of particular interest. Taking into consideration that OATP2B1 is expressed in certain human malignancies and it transports estrone-3-sulfate effectively, it would be worth designing, synthesizing and investigating potential 13α-estrone-based inhibitors against this transporter protein, too. Ring A substituents differing in position, size and polarity are expected to have remarkable influence on the affinity for the target proteins. Investigation of the influence of the nature of the atom attached directly to the aromatic ring A of 13α-estrone and the stereochemistry of the introduced moiety would also be of high interest.

**Scheme 1:**
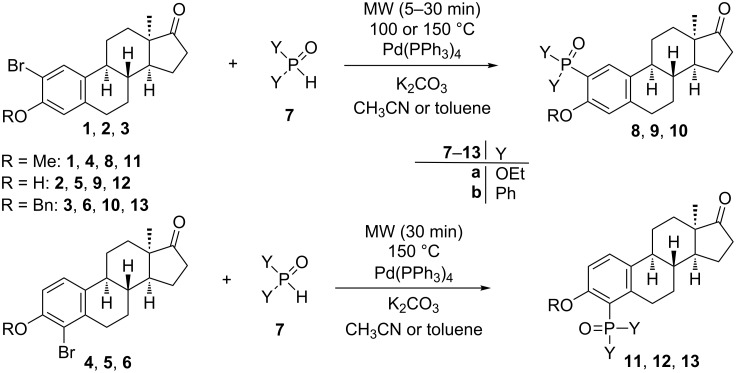
Pd-catalyzed C(sp^2^)–P couplings at C-2 or C-4 in the 13α-estrone series.

Here we disclose the synthesis of novel 2- or 4-substituted 13α-estrone derivatives **8**–**13** via the Hirao reaction (Scheme 1). Diethyl phosphite (**7a**) or diphenylphosphine oxide (**7b**) were chosen as >P(O)H reagents and C–P couplings were planned under microwave irradiation using Pd-based catalysis. Investigation of inhibitory activities of the newly-synthesized compounds **8**–**13** against the OATP2B1 transporter protein and 17β-HSD1 or STS cytosolic enzymes was also aimed.

## Results and Discussion

The Hirao reaction is a powerful tool for the synthesis of arylphosphonates from aryl bromides or iodides using dialkyl phosphites (H-phosphonates) as the reagents, Pd(PPh_3_)_4_ as the catalyst and Et_3_N as the base [26]. Variations of the reaction have recently been published to prepare diverse functionalized phosphonates [27–28]. The C–P couplings have been performed not only under traditional thermal conditions, but the important benefits of microwave-irradiation have also been utilized in this field [29].

The optimization of reaction conditions was carried out using 2-bromo- or 2-iodo-13α-estrone 3-methyl ether (**1** or **1I**) as starting compounds and diethyl phosphite (**7a**) as the reagent (Table 1, Scheme 1). Two Pd sources, namely Pd(PPh_3_)_4_ or Pd(OAc)_2_ were used, the latter without the addition of any mono- or bidentate P-ligand. Although Et_3_N can usually be used successfully, the dealkylation of the phosphite reagent may occur. In order to avoid this unwanted side reaction, K_2_CO_3_ was also involved in optimization. The selection of acetonitrile as solvent was based on literature data [27–29]. Acetonitrile is one of the most extensively used solvents in the Hirao reaction. Couplings were carried out under microwave irradiation. The quantity of diethyl phosphite was selected according to literature data [29]; namely, 1 equiv in the case of Pd(PPh_3_)_4_ and 1.3 equiv when using Pd(OAc)_2_. It was earlier established that in the microwave-assisted Pd(II)-catalyzed C–P coupling of aryl bromides and dialkyl phosphites, an excess of the applied dialkyl phosphite may serve as phosphorus ligand and reducing agent [29].

**Table 1:** Effect of the reaction conditions on Hirao reaction of 2-bromo- or 2-iodo-13α-estrone 3-methyl ether (**1** or **1I**) with diethyl phosphite (**7a**).

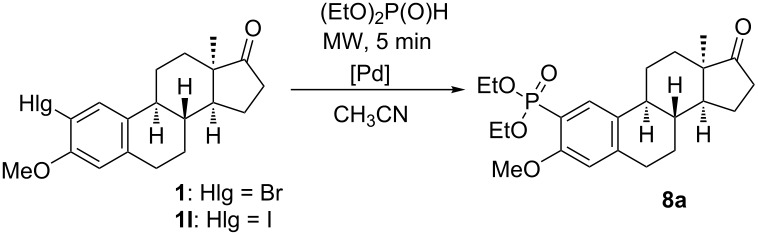						

entry	(EtO)_2_P(O)H quantity [equiv]	Hlg	Pd source [mol %]	base	temperature [°C]	yield^a^ [%]

1	1.3	Br	Pd(OAc)_2_ [10]	K_2_CO_3_	150	65
2	1.3	Br	Pd(OAc)_2_ [10]	K_2_CO_3_	100	77
3	1.3	I	Pd(OAc)_2_ [10]	K_2_CO_3_	150	61
4	1.3	I	Pd(OAc)_2_ [10]	K_2_CO_3_	100	66
5	1.3	Br	Pd(OAc)_2_ [10]	Et_3_N	150	58
6	1.3	Br	Pd(OAc)_2_ [10]	Et_3_N	100	59
7	1.3	I	Pd(OAc)_2_ [10]	Et_3_N	150	60
8	1.3	I	Pd(OAc)_2_ [10]	Et_3_N	100	62
9	1.0	Br	Pd(PPh_3_)_4_ [10]	K_2_CO_3_	150	73
10	1.0	Br	Pd(PPh_3_)_4_ [10]	K_2_CO_3_	100	89
11	1.0	I	Pd(PPh_3_)_4_ [10]	K_2_CO_3_	150	69
12	1.0	I	Pd(PPh_3_)_4_ [10]	K_2_CO_3_	100	72
13	1.0	Br	Pd(PPh_3_)_4_ [10]	Et_3_N	150	70
14	1.0	Br	Pd(PPh_3_)_4_ [10]	Et_3_N	100	78
15	1.0	I	Pd(PPh_3_)_4_ [10]	Et_3_N	150	66
16	1.0	I	Pd(PPh_3_)_4_ [10]	Et_3_N	100	70

^a^Obtained after flash chromatography.

The aryl bromide or iodide **1** or **1I** was dissolved in acetonitrile, the reagent, the catalyst and the base were added and then the mixture was irradiated in a microwave reactor for 5 min. The nature of the Pd source, the base, the reaction temperature and the halogen substantially influenced the yields of the C–P couplings. Reactions with catalyst precursor Pd(OAc)_2_ gave the desired phosphonate **8a** in moderate yields (Table 1, entries 1–8). Starting from 2-iodo compound **1I**, dehalogenation, an unwanted side reaction occurred, in particular, at elevated temperature (Table 1, entries 3, 4, 7, 8). Pd(PPh_3_)_4_, used as catalyst in the classical Hirao reaction [26], proved to be more efficient concerning the yields of the desired arylphosphonate (**8a**, Table 1, entries 9–16). K_2_CO_3_ as inorganic base seemed to be superior to organic Et_3_N (Table 1, entries 1–4, 9–12). The extent of dehalogenation could be suppressed by lowering the reaction temperature (Table 1, entries 2, 4, 6, 8, 10, 12, 14, 16). Conditions described in entry 10 proved to be optimal for coupling of compound **1** with diethyl phosphite (**7a**). These conditions were selected for extension of C–P couplings to other steroidal scaffolds **2**–**6**, which differ in regioisomerism and/or nature of the C-3 substituent. In order to get certain functionalized 13α-estrone derivatives with a different substitution pattern for biological investigations, not only 2-bromo **1**–**3**, but 4-bromo compounds **4**–**6** were also coupled with the two reagents **7a** and **7b** differing in size and polarity (Table 2, Scheme 1). In certain cases (Table 2, entries 2–6 and 8–12), however, it was necessary to change the optimal conditions (Table 1, entry 10) concerning reaction time and temperature. 2-Regioisomers **1**–**3** could successfully be transformed without significant changes in reaction conditions (Table 2, entries 1, 7), but the reaction time had to be prolonged in four cases (Table 2, entries 3, 5, 9, 11). However, in the reactions of 2-bromo 3-benzyl ether **3** and the 4-regioisomers **4**–**6** higher temperature (150 °C) was needed (Table 2, entries 2, 4, 5, 6, 8, 10, 11,12). The steric hindrance resulting from the presence of ring B near C-4 might be the main cause for the harsher reaction conditions. This is in agreement with our recently published Sonogashira coupling procedure, which required higher temperatures starting from 4-regioisomers in comparison with those applied for their 2-substituted counterparts [22]. Concerning the nature of the C-3 substituent, methyl ethers **1** and **4** were overally the most reactive. Compounds bearing a 3-OH group **2** and **5** reacted slower due to the more activated nature of the phenolic ring A. It can be stated that the reaction time and temperature greatly depends on the nature of the C-3 substituent and on the position of the bromine, not on the size and polarity of the P-coupling agent. In the case of 3-benzyl ethers **3** and **6**, solvent change from acetonitrile to toluene was required. This might be attributed to the decreased polarity of the 3-benzyloxy compounds **3** and **6**. We have recently disclosed our finding that toluene is an appropriate solvent for C(sp^2^)–N coupling reactions of 2- or 4-bromo-13α-estrone 3-benzyl ethers [30]. The structures of the newly-synthesized derivatives **8**–**13** were established through ^1^H, ^13^C, ^31^P, COSY, HSQC and/or HMBC measurements.

**Table 2:** Scope of the reaction^a^ of 2- or 4-bromo-13α-estrones **1**–**6** with diethyl phosphite (**7a**) or diphenylphosphine oxide (**7b**).

entry	substrate		>P(O)H	solvent	temp [°C]	reaction time [min]	product		yield^b^ [%]

1	**1**	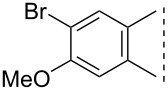	(EtO)_2_P(O)H	CH_3_CN	100	5	**8a**	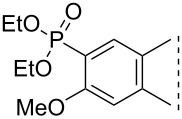	89
2	**4**	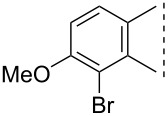	(EtO)_2_P(O)H	CH_3_CN	150	30	**11a**	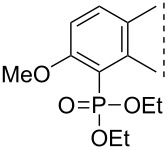	72
3	**2**	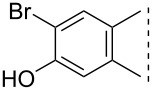	(EtO)_2_P(O)H	CH_3_CN	100	15	**9a**	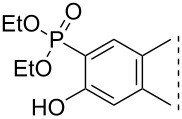	87
4	**5**	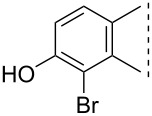	(EtO)_2_P(O)H	CH_3_CN	150	30	**12a**	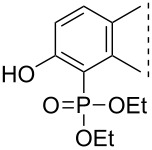	71
5	**3**	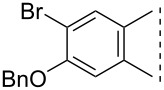	(EtO)_2_P(O)H	toluene	150	30	**10a**	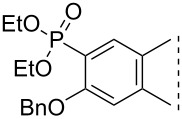	90
6	**6**	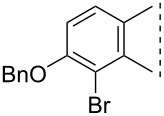	(EtO)_2_P(O)H	toluene	150	30	**13a**	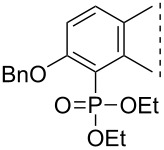	79
7	**1**	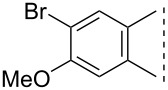	Ph_2_P(O)H	CH_3_CN	100	5	**8b**	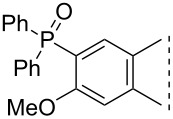	93
8	**4**	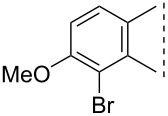	Ph_2_P(O)H	CH_3_CN	150	30	**11b**	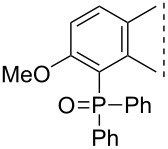	82
9	**2**	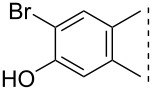	Ph_2_P(O)H	CH_3_CN	100	15	**9b**	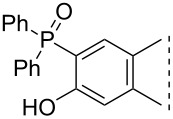	90
10	**5**	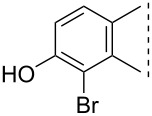	Ph_2_P(O)H	CH_3_CN	150	30	**12b**	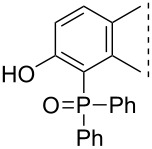	83
11	**3**	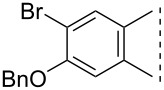	Ph_2_P(O)H	toluene	150	30	**10b**	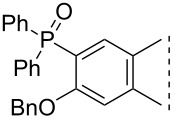	92
12	**6**	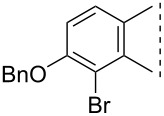	Ph_2_P(O)H	toluene	150	30	**13b**	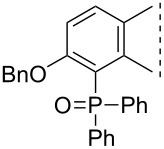	78

^a^Reactions were performed on a 0.25 mmol scale with 10 mol % Pd(PPh_3_)_4_ and K_2_CO_3_ (1.5 equiv) under microwave irradiation. ^b^Flash chromatography yields are reported.

The influence of the basic (13αE1OH, 13αE1OMe and 13αE1OBn) and new compounds **8**–**13** on the transport function of OATP2B1 was investigated by measuring Cascade Blue uptake (Table 3) [31]. 13α-Estrone (13αE1OH) itself displayed weak inhibitory action with an IC_50_ value above 50 μM. The presence of the methyl group at the phenolic OH function in compound 13αE1OMe increased inhibition dramatically. The 3-benzyl ether (13αE1OBn) exerted unexpected twofold inhibitory potency compared with that of its 3-methoxy counterpart 13αE1OMe. All newly-synthesized derivatives **8**–**13** displayed substantial inhibition on the transport function of OATP2B1, except for 4-substituted 3-OH compound **12b**. Derivatives **9a**, **9b** and **12a** bearing a 3-OH function and 2- or 4-substituted 3-MeO compounds **8** and **11** suppressed the transport in the low micromolar range. In the 3-OH series there appeared to be significant difference between the IC_50_ values of the potent derivatives **9a**, **9b** and **12a** and that of 13αE1OH. Furthermore, the inhibitory action of the basic reference compound 13αE1OMe was improved in compounds **8** and **11**. Surprisingly, but in accordance with the data obtained for compound 13αE1OBn, the most apolar 3-benzyl ethers **10** and **13** proved to be the best inhibitors with IC_50_ values in the submicromolar range. Accordingly, the inhibitory action of 13αE1OBn could be improved with one order of magnitude by the introduction of a diethyl phosphono group onto C-2 of 13αE1OBn. The most significant difference in IC_50_ values concerning the regioisomerism was observed among 3-OH test compounds **9b** and **12b** bearing the larger substituent. This might be attributed to certain intramolecular secondary interactions of the newly introduced substituent and the phenolic OH function, which may prevent the binding of the steroid to the transporter protein. Additionally, inhibitory potencies of the newly-synthesized compounds on human placental STS and 17β-HSD1 have been tested in vitro (Table 3). None of the newly-synthesized derivatives proved to be a potent STS inhibitor, since the compounds suppressed the estrone-sulfate to estrone conversion by less than 33% (applied in 10 μM test concentration). Henceforth we discuss the results of the 17β-HSD1 investigations. Compounds bearing phenolic OH function displayed the most potent inhibitory action, but exclusively the 2-regioisomers **9a** and **9b**. Introduction of a larger and less polar substituent onto C-2 seemed to be more advantageous, since compound **9b** displayed one order of magnitude lower IC_50_ value than that of its **9a** counterpart (IC_50_ values are 0.18 and 1.5 μM, respectively). A comparison of the IC_50_ values of compound **9b** and 13αE1OH indicates that introduction of a large diphenylphosphine oxide moiety onto C-2 improves the binding of the steroid to the enzyme. C-4-substituted 3-OH derivatives **12a** and **12b** barely suppressed the estrone to 17β-estradiol conversion. The same tendency showed up in our previous works regarding the inhibitory potentials of certain 2- or 4-halogenated or phenylalkynylated 13α-estrone derivatives [22–23]. Among 3-ethers, only the methyl ether analog of compound **9b** proved to be potent inhibitor with an IC_50_ value of 4.1 μM. The data obtained here for 3-methyl ethers do not correlate with those described earlier for 2- or 4-halogenated 3-MeO derivatives [21]. We established previously that 4-halo-3-methoxy derivatives are more potent 17β-HSD1 inhibitors than their 2-counterparts.

**Table 3:** OATP2B1, STS and 17β-HSD1 inhibition data of C–P coupled products **8**–**13** and their basic compounds 13αE1OMe, 13αE1OH, 13αE1OBn.

compound	OATP2B1 IC_50_ ± SD [μM]	STS rel. conv. ± SD^a^ [%]	17β-HSD1	
			
			IC_50_ ± SD [μM]	rel. conv. ± SD [%]

13αE1OMe	3.4 ± 0.3	99 ± 3	5.5 ± 1.5 [20]	99 ± 3
13αE1OH	>50	96 ± 1 [23]	1.2 ± 0.2 [20]	96 ± 1 [23]
13αE1OBn	1.7 ± 0.9	94 ± 3		94 ± 3
**8a**	1.8 ± 0.3	84 ± 4		60 ± 3
**11a**	2.8 ± 1.5	93 ± 7		100 ± 5
**9a**	2.1 ± 0.2	97 ± 5	1.5 ± 0.3	
**12a**	1.4 ± 0.1	93 ± 6		84 ± 4
**10a**	0.2 ± 0.02	85 ± 7		64 ± 3
**13a**	0.3 ± 0.02	87 ± 5		92 ± 3
**8b**	1.2 ± 0.1	80 ± 8	4.1 ± 1.1	
**11b**	2.5 ± 0.4	80 ± 5		106 ± 8
**9b**	2.6 ± 0.2	77 ± 10	0.18 ± 0.06	
**12b**	>50	93 ± 4		100 ± 5
**10b**	1 ± 0.1	85 ± 5		73 ± 3
**13b**	0.3 ± 0.02	105 ± 7		75 ± 2

^a^Mean ± standard deviation, *n* = 3.

## Conclusion

In conclusion, we have developed an efficient microwave-assisted Pd-catalyzed C–P coupling procedure for the synthesis of 2- or 4-phosphonated 13α-estrone derivatives **8**–**13**. The elaborated methodology proved to be suitable for the facile transformation of steroidal aryl bromides **1**–**6** containing OH, OMe or OBn functions in *ortho* positions. 2-Regioisomers of 3-OH derivatives **9a**,**b** appeared to be dual OATP2B1 and 17β-HSD1 inhibitors. The most selective compounds are the 3-benzyl ethers **10**, **13**, which inhibit only OATP2B1 from the three investigated targets. To the best of our knowledge, there are no literature reports concerning potent estrone-based OATP2B1 inhibitors.

## Author Contributions

The manuscript was written through contributions of all authors. All authors have given approval to the final version of the manuscript.

## Supporting Information

File 1 General synthetic procedures, characterization data for the synthesized compounds and biological assay methods.
