# Supplementary material for: Pd-Catalyzed microwave-assisted synthesis of phosphonated 13α-estrones as potential OATP2B1, 17β-HSD1 and/or STS inhibitors
Source: Beilstein J Org Chem. 2018 Nov 14;14:2838–45. doi: 10.3762/bjoc.14.262 (PMC6244214; doi:10.3762/bjoc.14.262)
Supplement: File 1 — General synthetic procedures, characterization data for the synthesized compounds and biological assay methods. [file Beilstein_J_Org_Chem-14-2838-s001.pdf]

BEILSTEIN JOURNAL OF ORGANIC CHEMISTRY

Supporting Information

for

Pd-Catalyzed microwave-assisted synthesis of phosphonated 13α-estrones as potential OATP2B1, 17β-HSD1 and/or STS inhibitors

Rebeka Jójárt, Szabolcs Pécsy, György Keglevich, Mihály Szécsi, Réka Rigó, Csilla Özvegy-Laczka, Gábor Kecskeméti and Erzsébet Mernyák

Beilstein J. Org. Chem. **2018,** *14,* 2838–2845. doi:10.3762/bjoc.14.262

General synthetic procedures, characterization data for the synthesized compounds and biological assay methods

License and Terms: This is a supporting information file under the terms of the Creative Commons Attribution License (http://creativecommons.org/licenses/by/4.0). Please note that the reuse, redistribution and reproduction in particular requires that the authors and source are credited.
The license is subject to the *Beilstein Journal of Organic Chemistry* terms and conditions: (https://www.beilstein-journals.org/bjoc)

Table of contents

EXPERIMENTAL S3
1. Chemistry S3
1.1. General S3
1.2. General procedure for microwave-assisted Hirao reaction of bromoestrone derivatives (**1**–**6**) S3
*1.2.1. Synthesis of diethyl (3-methoxy-13α-estra-1,3,5(10)-trien-17-on-2-yl)phosphonate (**8a**)* S4
*1.2.2. Synthesis of diethyl (3-methoxy-13α-estra-1,3,5(10)-trien-17-on-4-yl)phosphonate (**11a**)* S4
*1.2.3. Synthesis of diethyl (3-hydroxy-13α-estra-1,3,5(10)-trien-17-on-2-yl)phosphonate (**9a**)* S5
*1.2.4. Synthesis of diethyl (3-hydroxy-13α-estra-1,3,5(10)-trien-17-on-4-yl)phosphonate (**12a**)* S6
*1.2.5. Synthesis of diethyl (3-benzyloxy-13α-estra-1,3,5(10)-trien-17-on-2-yl)phosphonate (**10a**)* S6
*1.2.6. Synthesis of diethyl (3-benzyloxy-13α-estra-1,3,5(10)-trien-17-on-4-yl)phosphonate (**13a**)* S7
*1.2.7. Synthesis of (3-methoxy-13α-estra-1,3,5(10)-trien-17-on-2-yl)diphenylphosphine oxide (**8b**)* S8
*1.2.8. Synthesis of (3-methoxy-13α-estra-1,3,5(10)-trien-17-on-4-yl)diphenylphosphine oxide (**11b**)* S9
*1.2.9. Synthesis of (3-hydroxy-13α-estra-1,3,5(10)-trien-17-on-2-yl)diphenylphosphine oxide (**9b**)* S9
*1.2.10. Synthesis of (3-hydroxy-13α-estra-1,3,5(10)-trien-17-on-4-yl)diphenylphosphine oxide (**12b**)* S10
*1.2.11. Synthesis of (3-benzyloxy-13α-estra-1,3,5(10)-trien-17-on-2-yl)diphenylphosphine oxide (**10b**)* S11
*1.2.12. Synthesis of (3-benzyloxy-13α-estra-1,3,5(10)-trien-17-on-4-yl)diphenylphosphine oxide (**13b**)* S12
2. Transport assays S13
3. Enzymatic assays S13
3.1. General S13
3.2. Preparation of enzyme sources S13
3.3. Incubation procedures S14
3.4. Inhibition studies S14
REFERENCES S15

EXPERIMENTAL

1. Chemistry

1.1. General

Melting points (Mp) were determined with a Kofler hot-stage apparatus and are uncorrected. Elemental analyses were performed with a Perkin-Elmer CHN analyzer model 2400. Thin-layer chromatography: silica gel 60 F_{254}; layer thickness 0.2 mm (Merck); eluents: a: ethyl acetate, b: 10% ethyl acetate/90% dichloromethane, c: 50% ethyl acetate/50% dichloromethane, detection with I_2 or UV (365 nm) after spraying with 5% phosphomolybdic acid in 50% aqueous phosphoric acid and heating at 100–120 °C for 10 min. Flash chromatography: silica gel 60, 40–63 μm (Merck). Reactions under microwave irradiation were carried out with a CEM Corporation focused microwave system, Model Discover SP. The maximum power of irradiation was 200 W. ^{1}H NMR spectra were recorded in DMSO-d_6, $CDCl_3$ solution with a Bruker DRX-500 instrument at 500 MHz, with Me_4Si as internal standard. ^{13}C NMR spectra were recorded with the same instrument at 125 MHz under the same conditions. Mass spectrometry: Full scan mass spectra of the compounds were acquired in the range of 50 to 1000 *m/z* with a Finnigan TSQ-7000 triple quadrupole mass spectrometer (Finnigan-MAT, San Jose, CA) equipped with a equipped with a Finnigan electrospray ionization source. Analyses were performed in positive ion mode using flow injection mass spectrometry with a mobile phase of 50% aqueous acetonitrile containing 0.1% v/v formic acid. The flow rate was 0.3 ml/min. Five µl aliquot of the samples were loaded into the flow. The ESI capillary was adjusted to 4.5 kV and N_2 was used as a nebulizer gas.

1.2. General procedure for microwave-assisted Hirao reaction of bromoestrone derivatives 1–6)

2- or 4-Bromoestrone (**2** or **5**, 87 mg, 0.25 mmol) or 2- or 4-bromoestrone 3-methyl ether (**1** or **4**, 91 mg, 0.25 mmol) or 2- or 4-bromoestrone 3-benzyl ether (**3** or **6**, 110 mg, 0.25 mmol), tetrakis(triphenylphosphine)palladium(0) (28.9 mg, 0.025 mmol, 10 mol%), potassium carbonate (52 mg, 0.375 mmol, 1.5 equiv), diethyl phosphite (0.25 mmol, 34.5 mg) or diphenylphosphine oxide (0.25 mmol, 50.5 mg) and acetonitrile or toluene (3 ml) were added into a 10 ml Pyrex pressure vessel (CEM, Part #: 908035) with silicone cap (CEM, Part #: 909210). The mixture was irradiated in a CEM microwave reactor at 100 °C or 150 °C for 5–30 min under stirring. The solvent was evaporated in vacuo and the residue was purified by flash chromatography.

*1.2.1. Synthesis of diethyl (3-methoxy-13α-estra-1,3,5(10)-trien-17-on-2-yl)phosphonate (**8a**)*

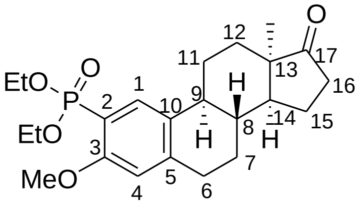

8a

As described in the general procedure, starting compound **1** (91 mg, 0.25 mmol) was reacted with diethyl phosphite (0.25 mmol, 34.5 mg) in acetonitrile (3 ml). The mixture was irradiated in a CEM microwave reactor at 100 °C for 5 min. After purification by flash chromatography with ethyl acetate as eluent, compound **8a** was obtained as a white solid (93 mg, 89%, Mp.: 129–130 °C, R_f = 0.44[a]). Anal calcd. for $C_{23}H_{33}O_5P$: C, 65.70; H, 7.91. Found: C, 65.77; H, 7.85. M_r: 420.21. ^{1}H NMR ($CDCl_3$) δ ppm: 1.05(s, 3H, 18-H_3); 1.30(t, 6H, *J*= 7.0 Hz, 2×OCH_2-C$\underline{H_3}$); 2.86(m, 2H, 6-H_2); 3.84(s, 3H, 3-OCH_3); 4.06−4.14(overlapping multiplets, 4H, 2×OC$\underline{H_2}$-CH_3); 6.61(d, 1H, *J*= 6.8 Hz, 4-H); 7.72(d, 1H, *J*= 15.6 Hz, 1-H). ^{13}C NMR δ ppm: 16.3 and 16.4(2×1C, 2×OCH_2−$\underline{C}H_3$); 20.9; 25.1(C-18); 27.9; 28.3; 30.8; 31.9; 33.4; 41.3; 41.4; 49.2; 50.1; 55.8(3-OCH_3); 61.9(2×1C, 2×O$\underline{C}H_2$-CH_3); 111.2(d, *J*= 9.9 Hz, C-4); 113.7 (d, *J*= 188.1 Hz, C-2); 131.7(d, *J*= 13.6 Hz, C-10); 133.2(d, *J*= 7.8 Hz, C-1); 143.7(d, *J*= 2.0 Hz, C-5); 159.0(C-3); 221.4 (C-17). ^{31}P NMR δ ppm: 18.2. MS *m/z* (%): 421 (100, $[M+H]^+$).

*1.2.2. Synthesis of diethyl (3-methoxy-13α-estra-1,3,5(10)-trien-17-on-4-yl)phosphonate (**11a**)*

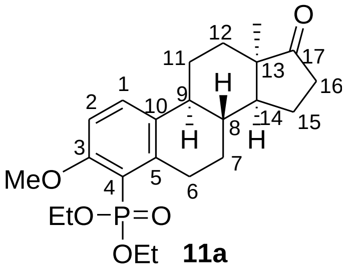

11a

As described in the general procedure, starting compound **4** (91 mg, 0.25 mmol) was reacted with diethyl phosphite (0.25 mmol, 34.5 mg) in acetonitrile (3 ml). The mixture was irradiated in a CEM microwave reactor at 150 °C for 30 min. After purification by flash chromatography with ethyl acetate as eluent, compound **11a** was obtained as a colorless oil (76 mg, 72%, R_f = 0.52[a]). Anal calcd. for $C_{23}H_{33}O_5P$: C, 65.70; H, 7.91. Found: C, 65.79; H, 7.84. M_r: 420.21. ^{1}H NMR ($CDCl_3$) δ ppm: 1.05(s, 3H, 18-H_3); 1.30−1.34(overlapping

multiplets, 6H, $2\times OCH_2\text{-}C\underline{H}_3$); 3.27(m, 2H, 6-$H_2$); 3.83(s, 3H, 3-$OCH_3$); 4.03−4.20(overlapping multiplets, 4H, $2\times OC\underline{H}_2\text{-}CH_3$); 6.77(dd, 1H, *J*= 8.8 Hz, *J*= 7.1 Hz, 2-H); 7.37(d, 1H, *J*= 8.8 Hz, 1-H). ^{13}C NMR δ ppm: 16.3 (d, *J*= 7.0 Hz, $OCH_2\text{-}\underline{C}H_3$); 16.4 (d, *J*= 7.2 Hz, $OCH_2\text{-}\underline{C}H_3$); 20.9; 24.9(C-18); 28.3; 28.6; 29.4(d, *J*= 3.4 Hz, C-6); 32.0; 33.4; 40.4; 41.7; 49.5; 50.2; 56.2(3-OCH_3); 61.4(d, *J*= 5.7 Hz, $O\underline{C}H_2\text{-}CH_3$); 61.7(d, *J*= 5.5 Hz, $O\underline{C}H_2\text{-}CH_3$); 109.5(d, *J*= 9.9 Hz, C-2); 114.8(d, *J*=183.0 Hz, C-4); 131.2(C-1); 133.7(d, *J*= 14.3 Hz, C-10); 144.6(d, 1C, *J*= 10.0 Hz, C-5); 160.3(C-3); 221.7(C-17). ^{31}P NMR δ ppm: 18.4. MS *m/z* (%): 354 (41); 421 (100, $[M+H]^+$).

*1.2.3. Synthesis of diethyl (3-hydroxy-13α-estra-1,3,5(10)-trien-17-on-2-yl)phosphonate (**9a**)*

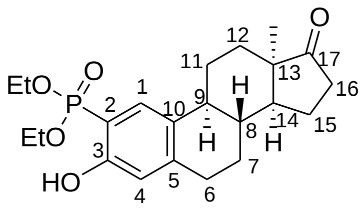

9a

As described in the general procedure, starting compound **2** (87 mg, 0.25 mmol) was reacted with diethyl phosphite (0.25 mmol, 34.5 mg) in acetonitrile (3 ml). The mixture was irradiated in a CEM microwave reactor at 100 °C for 15 min. After purification by flash chromatography with 10% ethyl acetate/90% dichloromethane as eluent, compound **9a** was obtained as a white solid (88 mg, 87%, Mp.: 139–140 °C, R_f =0.51[b]). Anal calcd. for $C_{22}H_{31}O_5P$: C, 65.01; H, 7.69. Found: C, 65.09; H, 7.62. M_r: 406.19. ^1H NMR ($CDCl_3$) δ ppm: 1.06(s, 3H, 18-H_3); 1.29(t, 3H, *J*= 7.1 Hz, $OCH_2\text{-}C\underline{H}_3$); 1.32(t, 3H, *J*= 7.1 Hz, $OCH_2\text{-}C\underline{H}_3$); 2.76−2.89(overlapping multiplets, 2H, 6-H_2); 3.93−4.06(overlapping multiplets, 2H, $OC\underline{H}_2\text{-}CH_3$); 3.07−4.18(overlapping multiplets, 2H, $OC\underline{H}_2\text{-}CH_3$); 6.68(d, 1H, *J*= 6.9 Hz, 4-H); 7.23(d, 1H, *J*= 15.2 Hz, 1-H); 9.95(s, 1H, OH). ^{13}C NMR δ ppm: 16.1(d, *J*= 6.2 Hz, $OCH_2\text{-}\underline{C}H_3$); 16.2(d, *J*= 6.0 Hz, $OCH_2\text{-}\underline{C}H_3$); 21.0; 25.0(C-18); 27.9; 28.0; 29.7; 30.3; 31.8; 33.4; 41.0; 41.1; 49.2; 50.1; 62.4(d, *J*= 4.6 Hz, $O\underline{C}H_2\text{-}CH_3$); 62.6(d, *J*= 4.6 Hz, $O\underline{C}H_2\text{-}CH_3$); 106.1(d, *J*= 181.4 Hz, C-2); 116.8(d, *J*= 12.4 Hz, C-4); 128.6(d, *J*= 7.1 Hz, C-1); 131.6(d, *J*= 13.5 Hz, C-10); 145.3(C-5); 159.7(d, *J*= 7.5 Hz, C-3); 221.5(C-17). ^{31}P NMR δ ppm: 23.0. MS *m/z* (%): 407 (100, $[M+H]^+$).

*1.2.4. Synthesis of diethyl (3-hydroxy-13α-estra-1,3,5(10)-trien-17-on-4-yl)phosphonate (**12a**)*

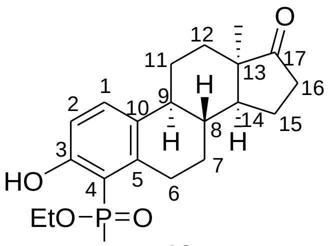

12a

As described in the general procedure, starting compound **5** (87 mg, 0.25 mmol) was reacted with diethyl phosphite (0.25 mmol, 34.5 mg) in acetonitrile (3 ml). The mixture was irradiated in a CEM microwave reactor at 150 °C for 30 min. After purification by flash chromatography with 10% ethyl acetate/90% dichloromethane as eluent, compound **12a** was obtained as a white solid (72 mg, 71%, Mp.: 170–171 °C, R_f = 0.69[b]). Anal calcd. for $C_{22}H_{31}O_5P$: C, 65.01; H, 7.69. Found: C, 65.08; H, 7.61. M_r: 406.19. ^{1}H NMR ($CDCl_3$) δ ppm: 1.05(s, 3H, 18-H_3); 1.31(t, 3H, *J*= 7.1Hz, OCH_2-C$\underline{H_3}$), 1.34(t, 3H, *J*= 7.1 Hz, OCH_2-C$\underline{H_3}$), 2.75 and 3.04(2×m, 2×1H, 6-H_2); 4.00–4.07(overlapping multiplets, 2H, OC$\underline{H_2}$-CH_3), 4.11–4.19(overlapping multiplets, 2H, OC$\underline{H_2}$-CH_3); 6.77(t, 1H, *J*= 7.1 Hz, 2-H); 7.35(d, 1H, *J*= 8.8 Hz, 1-H); 11.18(s, 1H, 3-OH). ^{13}C NMR δ ppm: 16.1(d, *J*= 6.6 Hz, OCH_2-$\underline{C}H_3$); 16.2(d, *J*= 6.6 Hz, OCH_2-$\underline{C}H_3$); 20.9; 25.0(C-18); 28.0; 28.4; 28.5(d, *J*= 4.4 Hz, C-6); 31.9; 33.3; 40.7; 41.6; 49.3; 50.1; 62.4(d, 2C, *J*= 5.3 Hz, O$\underline{C}H_2$-CH_3); 105.9(d, *J*= 174 Hz, C-4); 115.7(d, *J*= 13.3 Hz, C-2); 131.2 (d, *J*= 13.1 Hz, C-10); 132.9(d, *J*= 2.0 Hz, C-1); 140.7(d, *J*= 7.9 Hz, C-5); 161.7(d, *J*= 7.7 Hz, C-3); 221.4(C-17). ^{31}P NMR δ ppm: 25.3. MS *m/z* (%): 407 (100, $[M+H]^+$).

*1.2.5. Synthesis of diethyl (3-benzyloxy-13α-estra-1,3,5(10)-trien-17-on-2-yl)phosphonate (**10a**)*

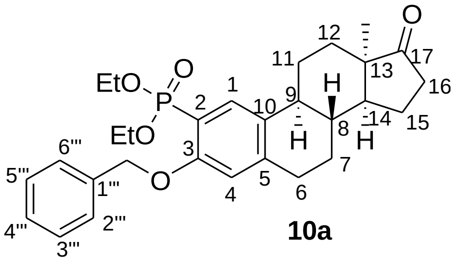

10a

As described in the general procedure, starting compound **3** (110 mg, 0.25 mmol) was reacted with diethyl phosphite (0.25 mmol, 34.5 mg) in toluene (3 ml). The mixture was irradiated in a CEM microwave reactor at 150 °C for 30 min. After purification by flash chromatography

with 50% ethyl acetate/50% dichloromethane as eluent, compound **10a** was obtained as a white solid (112 mg, 90%, Mp.: 104–105 °C, R_f = 0.62[c]). Anal calcd. for $C_{29}H_{37}O_5P$: C, 70.14; H, 7.51. Found: C, 70.22; H, 7.43. M_r: 496.24. ^{1}H NMR ($CDCl_3$) δ ppm: 1.05(s, 3H, 18-H_3); 1.25(t, 6H, *J*= 7.1 Hz, 2×OCH_2-C$\underline{H_3}$); 2.83(m, 2H, 6-H_2); 4.01–4.07(overlapping multiplets, 2H, OC$\underline{H_2}$-CH_3); 4.10–4.15(overlapping multiplets, 2H, OC$\underline{H_2}$-CH_3); 5.12(s, 2H, 3-OCH_2); 6.66(d, 1H, *J*= 6.7 Hz, 4-H); 7.31(t, 1H, *J*= 7.5 Hz, 4’”-H); 7.37(t, 2H, *J*= 7.5 Hz, 3’”-H and 5’”-H); 7.51(d, 2H, *J*= 7.5 Hz, 2’”-H and 6’”-H); 7.78(d, 1H, *J*= 15.8 Hz, 1-H). ^{13}C NMR δ ppm: 16.2(d, *J*= 2.1 Hz, OCH_2-$\underline{C}H_3$); 16.4(d, *J*= 2.1 Hz, OCH_2-$\underline{C}H_3$); 20.9; 25.1(C-18); 27.7; 28.3; 30.8; 31.9; 33.4; 41.3; 41.4; 49.2; 50.1; 61.9(d, 2C, 2×O$\underline{C}H_2$-CH_3); 70.0(3-OCH_2); 112.4(d, *J*= 9.9 Hz, C-4); 114.1(d, *J*= 188.9 Hz, C-2); 126.9(2C: C-3’” and C-5’”); 127.6(C-4’”); 128.4(2C: C-2’” and C-6’”); 132.1(d, *J*= 14.0 Hz, C-10); 133.4 (d, *J*= 8.1 Hz, C-1); 136.7(C-1’”); 143.7(C-5); 157.9(C-3); 221.3(C-17). ^{31}P NMR δ ppm: 18.1. MS *m/z* (%): 497 (100, $[M+H]^+$).

*1.2.6. Synthesis of diethyl (3-benzyloxy-13α-estra-1,3,5(10)-trien-17-on-4-yl)phosphonate **(13a)***

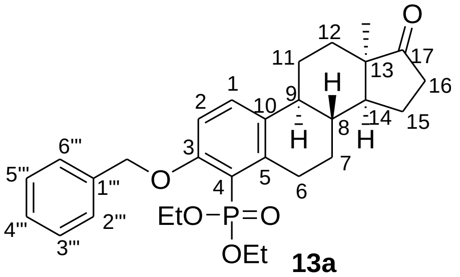

13a

As described in the general procedure, starting compound **6** (110 mg, 0.25 mmol) was reacted with diethyl phosphite (0.25 mmol, 34.5 mg) in toluene (3 ml). The mixture was irradiated in a CEM microwave reactor at 150 °C for 30 min. After purification by flash chromatography with 50% ethyl acetate/50% dichloromethane as eluent, compound **13a** was obtained as a colorless oil (98 mg, 79%, R_f = 0.62[c]). Anal calcd. for $C_{29}H_{37}O_5P$: C, 70.14; H, 7.51. Found: C, 70.20; H, 7.45. M_r: 496.24. ^{1}H NMR ($CDCl_3$) δ ppm: 1.04(s, 3H, 18-H_3); 1.22(td, 6H, *J*= 7.1 Hz, *J*= 1.3 Hz, 2×OCH_2-C$\underline{H_3}$); 3.32(m, 2H, 6-H_2); 3.99–4.06(overlapping multiplets, 2H, OC$\underline{H_2}$-CH_3); 4.07–4.16(overlapping multiplets, 2H, OC$\underline{H_2}$-CH_3); 5.10(s, 2H, 3-OCH_2); 6.81(dd, 1H, *J*= 8.2 Hz, *J*= 6.8 Hz, 2-H); 7.30(t, 1H, *J*= 7.4 Hz, 4’”-H); 7.34−7.38(overlapping multiplets, 3H, 1-H and 3’”-H and 5’”-H); 7.47(d, 2H, *J*= 7.4 Hz, 2’”-H, 6’”-H). ^{13}C NMR δ ppm: 16.2(d, *J*= 2.0 Hz, OCH_2-$\underline{C}H_3$); 16.3(d, *J*= 2.5 Hz, OCH_2-$\underline{C}H_3$); 20.9; 24.9(C-18); 28.2; 28.6; 29.3(d, *J*= 3.1 Hz, C-6); 32.0; 33.4; 40.3; 41.7; 49.5; 50.1;

61.4(d, *J*= 5.5 Hz, O<u>C</u>H_2-CH_3); 61.6(d, *J*= 5.5 Hz, O<u>C</u>H_2-CH_3); 70.8(3-OCH_2); 110.6(d, *J*= 10.3 Hz, C-2); 115.0(d, *J*= 183.1 Hz, C-4); 127.2(2C: C-3"' and C-5"'); 127.7(C-4"'); 128.4(2C: C-2"' and C-6"'); 131.2(C-1); 134.0(d, *J*= 15.0 Hz, C-10); 136.7(C-1"'); 144.8(d, *J*= 11.0 Hz, C-5); 159.4(C-3); 221.7(C-17). ^{31}P NMR δ ppm: 18.3. MS *m/z* (%): 497 (100, $[M+H]^+$).

1.2.7. Synthesis of (3-methoxy-13α-estra-1,3,5(10)-trien-17-on-2-yl)diphenylphosphine oxide ***(8b)***[a]

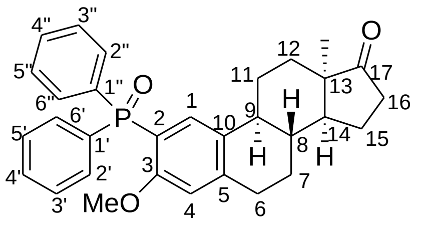

8b

As described in the general procedure, starting compound **1** (91 mg, 0.25 mmol) was reacted with diphenylphosphine oxide (0.25 mmol, 50.5 mg) in acetonitrile (3 ml). The mixture was irradiated in a CEM microwave reactor at 100 °C for 5 min. After purification by flash chromatography with ethyl acetate as eluent, compound **8b** was obtained as a white solid (113 mg, 93%, Mp.: 154–155 °C, R_f = 0.52[a]). Anal calcd. for $C_{31}H_{33}O_3P$: C, 76.84; H, 6.86. Found: C, 76.91; H, 6.80. M_r: 484.22. ^{1}H NMR ($CDCl_3$) δ ppm: 1.04(s, 3H, 18-H_3); 2.88(m, 2H, 6-H_2); 3.49(s, 3H, 3-OCH_3); 6.60(d, 1H, *J*= 5.4 Hz, 4-H); 7.39−7.44(overlapping multiplets, 4H, 3'-, 3"-, 5'- and 5"-H); 7.47−7.52(overlapping multiplets, 2H, 4'-H and 4"-H); 7.66−7.72(overlapping multiplets, 4H, 2'-, 2"-, 6'- and 6"-H); 7.73(d, 1H, *J*=10.8 Hz, 1-H). ^{13}C NMR δ ppm: 20.9; 25.0(C-18); 27.9; 28.3; 30.8; 31.9; 33.4; 41.4; 41.5; 49.1; 50.1; 55.2(3-OCH_3); 111.5(d, *J*= 7.0 Hz, C-4); 116.7(d, *J*= 107.0 Hz, C-2); 128.0(d, 2C, *J*= 12.3 Hz, C-3' and C-5'); 128.1(d, 2C, *J*= 12.3 Hz, C-3" and C-5"); 131.5(d, *J*= 2.3 Hz, C-4'); 131.6(d, *J*= 2.3 Hz, C-4"); 131.8(d, 2C, *J*= 10.2 Hz, C-2' and C-6'); 131.9(d, 2C, *J*= 10.2 Hz, C-2" and C-6"); 132.6(d, *J*= 11.3 Hz, C-10); 132.9(d, *J*= 7.5 Hz, C-1); 143.9(C-5); 158.5(d, *J*= 3.5 Hz, C-3); 221.4(C-17). ^{31}P NMR δ ppm: 30.1. MS *m/z* (%): 485 (100, $[M+H]^+$).

*1.2.8. Synthesis of (3-methoxy-13α-estra-1,3,5(10)-trien-17-on-4-yl)diphenylphosphine oxide (**11b**)*[a]

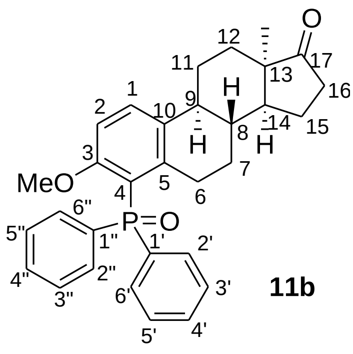

11b

As described in the general procedure, starting compound **4** (91 mg, 0.25 mmol) was reacted with diphenylphosphine oxide (0.25 mmol, 50.5 mg) in acetonitrile (3 ml). The mixture was irradiated in a CEM microwave reactor at 150 °C for 30 min. After purification by flash chromatography with ethyl acetate as eluent, compound **11b** was obtained as a white solid (99 mg, 82%, Mp: 97–98 °C, Rf = 0.58[a]). Anal calcd. for $C_{31}H_{33}O_3P$: C, 76.84; H, 6.86. Found: C, 76.93; H, 6.79. M_r: 484.22. ^{1}H NMR ($CDCl_3$) δ ppm: 1.05(s, 3H, 18-H_3); 3.09(s, 3H, 3-OCH_3); 3.27 and 3.47(2×m, 2×1H, 6-H_2); 6.67(dd, 1H, *J*= 8.5 Hz, *J*= 5.1 Hz, 2-H); 7.37−7.46(overlapping multiplets, 6H, 1-H, 3'-, 4'-, 5'-, 3"-, 5"-H); 7.50(m, 1H, 4"-H); 7.58(m, 2H, 2'-H and 6'-H); 7.74(m, 2H, 2"-H and 6"-H). ^{13}C NMR δ ppm: 20.9; 24.9(C-18); 28.1; 28.6; 28.9(d, 1C, *J*= 4.0 Hz, C-6); 32.0; 33.3; 40.3; 41.7; 49.4; 50.2; 54.8(3-OCH_3); 109.5(d, *J*= 7.0 Hz, C-2); 116.9(d, *J*= 107.0 Hz, C-4); 128.0(d, 2C, *J*= 12.5 Hz, C-3' and C-5'); 128.1(d, 2C, *J*= 12.5 Hz, C-3", C-5"); 130.7(d, 2C, *J*=10.0 Hz, C-2' and C-6'); 130.8(m, C-4'); 131.2(m, C-4"); 131.3 (d, 2C, *J*= 10.5 Hz, C-2" and C-6"); 131.5(C-1); 134.6(d, 1C, *J*= 10.1 Hz, C-10); 136.4(d, 2C, *J*= 109.3 Hz, C-1' and C-1"), 146.4(d, 1C, *J*= 6.4 Hz, C-5); 158.9(d, 1C, *J*= 4.2 Hz, C-3); 221.8 (C-17). ^{31}P NMR δ ppm: 31.7. MS *m/z* (%): 485 (100, $[M+H]^+$).

*1.2.9. Synthesis of (3-hydroxy-13α-estra-1,3,5(10)-trien-17-on-2-yl)diphenylphosphine oxide (**9b**)*[a]

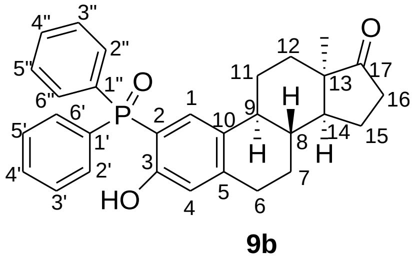

9b

As described in the general procedure, starting compound **2** (87 mg, 0.25 mmol) was reacted with diphenylphosphine oxide (0.25 mmol, 50.5 mg) in acetonitrile (3 ml). The mixture was irradiated in a CEM microwave reactor at 100 °C for 15 min. After purification by flash chromatography with 10% ethyl acetate/90% dichloromethane as eluent, compound **9b** was obtained as a white solid (106 mg, 90%, Mp.: 201–202 °C, Rf = 0.36[b]). Anal calcd. for $C_{30}H_{31}O_3P$: C, 76.58; H, 6.64. Found: C, 76.64; H, 6.58. M_r: 470.20. ^{1}H NMR ($CDCl_3$) δ ppm: 1.02(s, 3H, 18-H_3); 2.84(m, 2H, 6-H_2); 6.68(d, 1H, *J*= 4.5 Hz, 4-H); 6.83(d, 1H, *J*= 14.0 Hz, 1-H); 7.47–7.50(overlapping multiplets, 4H, 3'-, 3"-, 5'- and 5"-H); 7.58–7.60(overlapping multiplets, 2H, 4'-H and 4"-H); 7.63–7.69(overlapping multiplets, 4H, 2'-, 2"-, 6' and 6"-H). ^{13}C NMR δ ppm: 20.9; 24.9(C-18); 27.8; 28.0; 30.2; 31.7; 33.3; 40.9; 41.1; 49.1, 50.1; 108.2(d, *J*= 106.6 Hz, C-2); 117.8(d, *J*= 8.3 Hz, C-4); 128.6(d, 2C, *J*= 3.3 Hz, C-3' and C-5'); 128.7(d, 2C, *J*= 3.3 Hz, C-3" and C-5"); 129.1(d, *J*= 10.6 Hz, C-1); 130.9(d, *J*= 11.8 Hz, C-10); 131.7(d, 2C, *J*= 10.5 Hz, C-2' and C-6'); 131.8(d, *J*= 104.4 Hz, C-1'); 131.9(d, 2C, *J*= 10.6 Hz, C-2" and C-6"); 132.1(d, *J*= 102.1 Hz, C-1"); 132.3(d, *J*= 2.8 Hz, C-4'); 132.5(d, *J*= 2.8 Hz, C-4"); 144.2(d, *J*= 2.2 Hz, C-5); 161.5(d, *J*= 3.1 Hz, C-3); 221.7(C-17). MS *m/z* (%): 471 (100, $[M+H]^+$).

1.2.10. Synthesis of (3-hydroxy-13α-estra-1,3,5(10)-trien-17-on-4-yl)diphenylphosphine oxide ***(12b)***[a]

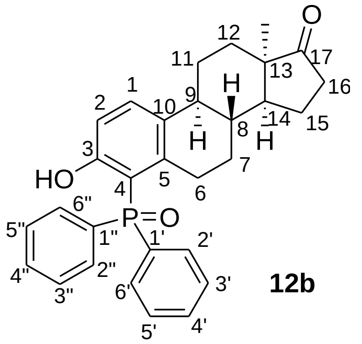

12b

As described in the general procedure, starting compound **5** (87 mg, 0.25 mmol) was reacted with diphenylphosphine oxide (0.25 mmol, 50.5 mg) in acetonitrile (3 ml). The mixture was irradiated in a CEM microwave reactor at 150 °C for 30 min. After purification by flash chromatography with 10% ethyl acetate/90% dichloromethane as eluent, compound **12b** was obtained as a white solid (98 mg, 83%, Mp.: 264−267°C, R_f = 0.56[b]). Anal calcd. for $C_{30}H_{31}O_3P$: C, 76.58; H, 6.64. Found: C, 76.63; H, 6.59. M_r: 470.20. ^{1}H NMR ($CDCl_3$) δ ppm: 1.00(s, 3H, 18-H_3), 6.87(dd, 1H; *J*= 8.8 Hz, *J* = 4.6 Hz, 2-H); 7.37(d, 1H, *J*= 8.8 Hz, 1-

H); 7.49–7.54(overlapping multiplets, 4H, 3’-, 3”-, 5’ and 5”-H); 7.57–7.63(overlapping multiplets, 2H, 4’-H and 4”-H); 7.71–7.75(overlapping multiplets, 4H, 2’-, 2”-, 6’- and 6”-H). ^{13}C NMR (DMSO-d_6) δ ppm: 20.3; 24.3(C-18); 27.1; 27.7; 29.4(d, *J*= 5.3 Hz, C-6); 31.4; 32.6; 40.4; 48.8(2C); 49.4; 108.8(d, *J*= 100.3 Hz, C-4); 115.4(d, *J*= 7.9 Hz, C-2); 128.7 (d, 4C, *J*= 12.0 Hz, C-3’, -3”, -5’ and -5”); 131.0 (d, 2C, *J*= 10.6 Hz, C-2’ and C-6’); 131.1 (d, 2C, *J*= 10.5 Hz, C-2” and C-6”); 131.6 (d, *J*= 9.9 Hz, C-10); 131.9 (C-1); 132.1 (2C, C-4’and C-4”); 132.8 (d, *J*= 104.5 Hz, C-1’); 133.1 (d, *J*= 104.7 Hz, C-1”); 141.6 (d, *J*= 8.5 Hz, C-5); 161.3 (d, *J*= 4.1 Hz, C-3); 220.5 (C-17). ^{31}P NMR δ ppm: 43.6. MS *m/z* (%): 471 (100, $[M+H]^+$).

*1.2.11. Synthesis of (3-benzyloxy-13α-estra-1,3,5(10)-trien-17-on-2-yl)diphenylphosphine oxide (**10b**)*[a]

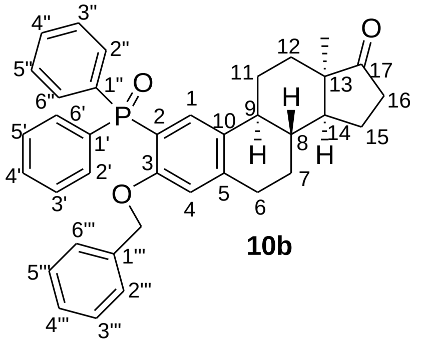

10b

As described in the general procedure, starting compound **3** (110 mg, 0.25 mmol) was reacted with diphenylphosphine oxide (0.25 mmol, 50.5 mg) in toluene (3 ml). The mixture was irradiated in a CEM microwave reactor at 150 °C for 30 min. After purification by flash chromatography with 50% ethyl acetate/50% dichloromethane as eluent, compound **10b** was obtained as a white solid (129 mg, 92%, Mp.: 255–256 °C, R_f = 0.53[c]). Anal calcd. for $C_{37}H_{37}O_3P$: C, 79.26; H, 6.65. Found: C, 79.32; H, 6.58. M_r: 560.25. ^{1}H NMR ($CDCl_3$) δ ppm: 1.05(s, 3H, 18-H_3); 2.85(m, 2H, 6-H_2); 4.79(s, 2H, 3-OCH_2); 6.62(d, 1H, *J*= 5.6 Hz, 4-H); 6.79(d, 2H, *J*= 7.3 Hz, 2”’-H and 6”’-H); 7.17(t, 2H, *J*=7.3 Hz, 3”’-H and 5”’-H); 7.23(t, 1H, *J*=7.3 Hz, 4”’-H); 7.29–7.36(overlapping multiplets, 4H, 3’-, 3”-, 5’- and 5”-H); 7.44–7.49(overlapping multiplets, 2H, 4’-H and 4”-H); 7.59–7.67(overlapping multiplets, 4H, 2’-, 2”-, 6’- and 6”-H); 7.91(d, 1H, *J*=14.1 Hz, 1-H). ^{13}C NMR δ ppm: 20.9; 25.1(C-18); 27.8; 28.4; 30.8; 31.9; 33.4; 41.4; 41.5; 49.2; 50.1; 70.0(3-OCH_2); 111.8(d, *J*= 7.0 Hz, C-4); 117.1(d, *J*= 105.4, C-2); 127.5(2C: C-2”’ and C-6”’); 127.8(C-4”’); 127.9(d, 2C, *J*=15.2 Hz, C-3’ and C-5’); 128.0(d, 2C, *J*=15.4 Hz, C-3” and C-5”); 128.2(2C: C-3”’ and C-5”’);

131.3(C-4’); 131.4(C-4”); 131.9 (d, 2C, *J*= 10.6 Hz, C-2’ and C-6’); 132.0(d, 2C, *J*= 10.4 Hz, C-2” and C-6”); 132.6(d, *J*= 104.5 Hz, C-1’); 132.7(d, *J*= 10.8 Hz, C-10); 133.0(d, *J*= 6.9 Hz, C-1); 133.1(d, *J*= 107.1 Hz, C-1”); 135.7(C-1’”); 143.7(C-5); 157.2(d, *J*= 4.1 Hz, C-3); 221.4(C-17). ^{31}P NMR δ ppm: 28.2. MS *m/z* (%): 561 (100, $[M+H]^+$).

*1.2.12. Synthesis of (3-benzyloxy-13α-estra-1,3,5(10)-trien-17-on-4-yl)diphenylphosphine oxide (**13b**)*[a]

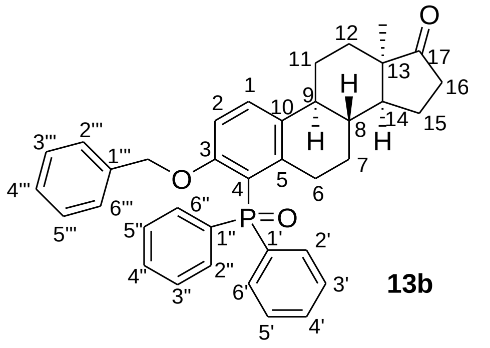

13b

As described in the general procedure, starting compound **6** (110 mg, 0.25 mmol) was reacted with diphenylphosphine oxide (0.25 mmol, 50.5 mg) in toluene (3 ml). The mixture was irradiated in a CEM microwave reactor at 150 °C for 30 min. After purification by flash chromatography with 50% ethyl acetate/50% dichloromethane as eluent, compound **13b** was obtained as a white solid (109 mg, 78%, Mp.: 74–75 °C, R_f = 0.56[c]). Anal calcd. for $C_{37}H_{37}O_3P$: C, 79.26; H, 6.65. Found: C, 79.33; H, 6.59. M_r: 560.25. ^{1}H NMR ($CDCl_3$) δ ppm: 1.04(s, 3H, 18-H_3); 3.38 and 3.60(2xm, 2x1H, 6-H_2); 4.40 and 4.54(2xd, 2x1H, *J*= 12.0 Hz, 3-OCH_2); 6.67(dd, 1H, *J*= 8.6 Hz, *J*= 5.6 Hz, 2-H); 6.74(d, 2H, *J*= 7.3 Hz, 2’”-H and 6’”-H); 7.17(t, 2H, *J*=7.3 Hz, 3’”-H and 5’”-H); 7.22(t, 1H, *J*= 7.3 Hz, 4’”-H); 7.28(m, 2H, 3’-H and 5’-H); 7.35–7.41(overlapping multiplets, 4H, 1-, 4’-, 3”- and 5”-H); 7.47(m, 1H, 4”-H); 7.51(dd, 2H, *J*= 12.6 Hz, *J*= 7.3 Hz, 2’-H and 6’-H); 7.68(dd, 2H, *J*= 12.6 Hz, *J*= 7.3 Hz, 2”-H and 6”-H). ^{13}C NMR δ ppm: 20.9; 24.9(C-18); 28.2; 28.6; 28.7(d, *J*= 3.5 Hz, C-6); 32.0; 33.4; 40.2; 41.7; 49.5; 50.1; 70.2(3-OCH_2); 109.8(C-2); 116.6(d, 1C, *J*= 101.9 Hz, C-4); 127.4(2C: C-2’” and C-6’”); 127.8(C-4’”); 128.0(d, 2C, *J*=15.5 Hz, C-3’ and C-5’); 128.1(d, 2C, *J*= 15.3 Hz, C-3” and C-5”), 128.3(2C: C-3’” and C-5’”); 130.7(d, 1C, *J*= 2.1 Hz, C-4’), 131.0(d, 2C, *J*= 10.1 Hz, C-2’ and C-6’); 131.1(d, 1C, *J*= 2.1 Hz, C-4”); 131.4(C-1); 131.5(d, 2C, *J*=10.3 Hz, C-2” and C-6”); 134.4(d, 1C, *J*= 106.5 Hz, C-1’); 134.7(d, 1C, *J*=10.3 Hz, C-10); 135.8(C-1’”); 135.9(d, 1C, *J*= 106.9 Hz, C-1”); 146.8(d, 1C, *J*= 6.5 Hz, C-5); 158.2(d, 1C, *J*= 4.3 Hz, C-3); 221.8(C-17). ^{31}P NMR δ ppm: 32.5. MS *m/z* (%): 561 (100, $[M+H]^+$).

[a] NMR data of the two phenyl rings originated from diphenyl phosphine oxide may be in reversed order.

2. Transport assays

The effect of the newly synthesized derivatives **8**–**13** and their basic compounds (13αE1OH, 13αE1OMe and 13αE1OBn) on OATP2B1 transport activity was determined by the CascadeBlue assay.[S1] A431 cells (8×10^4 in 200 µl) overexpressing human OATP2B1 or transfected with the vector control were seeded on 96 well plates 16 hours prior to the transport measurement. Transport reaction was performed as described in.[S1] Briefly, after washing the cells 3-times with 200 µl 1 × phosphate buffered saline (PBS) the cells were preincubated with test compounds in final concentrations from 0.03 to 50 µM or dimethyl sulfoxide (used as a solvent for the test compounds and serving as non-treated control) for 5 minutes at 37 °C. The reaction was started with the addition of 50 µl Cascade Blue (resulting in final concentration of 10 µM) and the cells were further incubated at 37 °C for 30 minutes. The reaction was stopped by the addition of 200 µl ice-cold 1 × PBS, and after 3 washing steps the fluorescence remaining in the cells was determined by Enspire fluorescent plate reader (Perkin Elmer) at 400 nm excitation and 419 nm emission wavelengths. Transport activity was determined by subtracting fluorescence in mock transfected cells. Kinetics of inhibition was determined by Hill fit using the Origin 8.6 software.

3. Enzymatic assays

3.1. General

The ability of the synthesized compounds **8**–**13** to inhibit activity of steroid sulfatase (STS) and 17β-hydroxysteroid dehydrogenase type 1 (17β-HSD1) was examined. In vitro radiosubstrate incubations were performed with adapted literature methods[S2–4] and human placenta served as source of the enzymes. Radiolabelled steroids were obtained from American Radiolabeled Chemicals (St. Louis, MO, USA). Other chemicals and solvents of analytical grade purity were purchased from Sigma (St. Louis, MO, USA), from Fluka (Buchs, Switzerland) or from Merck (Darmstadt, Germany).

3.2. Preparation of enzyme sources

Human term placentae were collected immediately after delivery and stored frozen at −80 °C. Tissue specimens were homogenized with an Ultra-Turrax in 0.1 M HEPES buffer solution

(pH 7.3) containing 1 mM EDTA and 1 mM dithiotreitol, and cytosol and microsomas were obtained with fractionated centrifugation. Application of the human tissue was approved by the institutional Human Investigation Review Board.

3.3. Incubation procedures

Enzymatic incubations were carried out in the HEPES buffer medium at a final volume of 200 µl. The substrate was added to the incubate in 20 µl of a 25 % v/v propylene glycol in HEPES buffer solution, whereas test compounds were applied in 10 µl of dimethyl sulfoxide solution. Incubations were performed at 37 °C and terminated by cooling and the addition of organic solvents of the subsequent extraction procedure. Control samples with no inhibitor and blank samples were incubated simultaneously. The radioactivity of the products formed was measured by means of liquid scintillation counting (LSC) and taken as indicator of the enzyme activity.

STS assays were performed using microsoma suspension and estrone-3-sulfate (E1S) substrate with [6,7-^{3}H]estrone-3-sulfate tracer with incubations lasting for 20 min. Reactions were terminated by the addition of equal volume of methanol, and the product estrone (E1) was extracted with toluene.[S4]

In the 17β-HSD1 assays the cytosol was incubated using estrone (E1) substrate with [6,7-^{3}H]estrone tracer and 0.1 mM NADPH cofactor excess. The incubation time was 2.5 min. The product 17β-estradiol (E2) was extracted with ethyl acetate and isolated by a TLC method.[S5]

3.4. Inhibition studies

In the general procedure, incubations were performed as described. Concentrations of the substrates were 1.0 µM, and the test compounds were applied at 10 µM final concentration in the incubate. Relative conversions compared to non-inhibited controls (100%) were determined. The assays were performed in triplicate, and the mean value and the standard deviation (SD) were calculated.

IC_{50} values (the inhibitor concentration that decreases the enzyme activity to 50%) were determined for the most effective test compounds. In these cases, conversions were measured at 10–15 different concentrations in the appropriate interval between 0.001–50 µM. IC_{50} results were calculated by using unweighted iterative least squares logistic curve fitting by means of the “absolute IC_{50} calculation“ function of the GraphPad Prism 4.0 software (GraphPad Software, Inc., San Diego, CA, USA).

REFERENCES

S1 Patik, I.; Székely, V., Német, O.; Szepesi, Á.; Kucsma, N.; Várady, G.; Szakács, G.; Bakos, É.; Özvegy-Laczka, C. Identification of novel cell-impermeant fluorescent substrates for testing the function and drug interaction of Organic Anion-Transporting Polypeptides, OATP1B1/1B3 and 2B1. *Sci Rep*. **2018**, *8*, 1–12.

S2 Leslie, K. K.; Zuckerman, D. J.; Schruefer, J.; Burchell, M.; Smith, J.; Albertson, B. D. Oestrogen modulation with parturition in the human placenta. *Placenta* **1994**, *15*, 79–88.

S3 Tremblay, M. R.; Poirier, D. Overview of a rational approach to design type I 17beta-hydroxysteroid dehydrogenase inhibitors without estrogenic activity: chemical synthesis and biological evaluation. *J. Steroid Biochem. Mol. Biol.* **1998**, *66*, 179–191.

S4 Bacsa, I.; Herman, B. E.; Jójárt R.; Herman, K. S.; Wölfling, J.; Schneider, G.; Varga, M.; Tömböly, C.; Lanisnik-Rizner, T.; Szécsi, M.; Mernyák, E. Synthesis and structure–activity relationships of 2- and/or 4-halogenated 13β- and 13α-estrone derivatives as enzyme inhibitors of estrogen biosynthesis. *J. Enzyme Inhib. Med. Chem.* **2018**, 10.1080/14756366.2018.1490731.

S5 Herman, B. E.; Szabó, J.; Bacsa, I.; Wölfling, J.; Schneider, G.; Bálint, M.; Hetényi, C.; Mernyák, E.; Szécsi, M. Comparative investigation of the in vitro inhibitory potencies of 13-epimeric estrones and D-secoestrones towards 17β-hydroxysteroid dehydrogenase type 1. *J. Enzyme Inhib. Med. Chem.* **2016**, *31*, 61–69.
